# Microfluidic Fabrication of Alendronate-Modified Lipid Nanoparticles for Bone-Targeted mRNA Delivery

**DOI:** 10.3390/pharmaceutics18040509

**Published:** 2026-04-20

**Authors:** Kangling Xu, Junyu Su, Hailin Ma, Yanxia Zhu

**Affiliations:** 1 School of Pharmacy, Shenzhen University Medical School, Shenzhen University, Shenzhen 518055, China; 2 School of Basic Medical Sciences, Shenzhen University Medical School, Shenzhen University, Shenzhen 518055, China

**Keywords:** lipid nanoparticles, bone targeting, alendronate, mRNA delivery, post-conjugation method, microfluidics

## Abstract

**Background/Objectives:** Bone-targeted drug delivery systems hold great promise for treating skeletal diseases, yet the optimal strategy for functionalizing lipid nanoparticles (LNPs) with bone-homing ligands remains insufficiently explored. Herein, we compared two alendronate sodium (Alen) modification approaches (pre-conjugation and post-conjugation) for constructing bone-targeted LNPs capable of delivering mRNA to skeletal tissues. **Methods:** LNPs were fabricated via microfluidic mixing, and the 1,2-distearoyl-sn-glycero-3-phosphoethanolamine-polyethylene glycol-alendronate conjugate (DSPE-PEG-Alen) required for the pre-conjugation method was synthesized. The bone-targeting ability of LNPs prepared by the two Alen modification strategies was evaluated using an *in vitro* hydroxyapatite (HAP) binding assay. Furthermore, the physicochemical properties, bone-targeting performance, mRNA delivery efficiency, and biosafety of the LNPs prepared by the post-conjugation method were assessed through cellular uptake, *in vivo* imaging, and other methods. **Results:** Hydroxyapatite binding assays revealed that the post-conjugation strategy afforded significantly superior bone affinity compared to the pre-conjugation approach. In addition, ex vivo bone fragment binding experiments further confirmed that the bone-targeting LNPs prepared by the post-conjugation method exhibited stronger bone-binding capability compared to unmodified LNPs. The optimized Alen-LNPs demonstrated efficient cellular uptake and functional mRNA translation in bone marrow mesenchymal stem cells with negligible cytotoxicity. *In vivo* studies in mice confirmed the preferential accumulation of Alen-LNPs in bone tissues, with successful green fluorescent protein (GFP) mRNA translation detected in bone tissue sections. Histopathological analysis confirmed the biosafety of the formulation. **Conclusions:** This study establishes the post-conjugation strategy as the superior approach for Alen functionalization of LNPs, providing a robust and reproducible platform for bone-targeted mRNA therapeutics.

## 1. Introduction

Skeletal system diseases, such as osteoporosis, bone tumors, and bone metastases, have become significant public health issues severely affecting human health [1]. Globally, approximately 200 million people suffer from osteoporosis, which leads to fractures that are among the leading causes of disability and mortality in the elderly population [2]. Moreover, the incidence of bone metastases exceeds 70% in patients with advanced-stage cancers, severely impacting patients’ quality of life and prognosis [3]. The treatment of these diseases typically requires maintaining effective drug concentrations at the lesion sites over prolonged periods. However, traditional systemic administration suffers from inherent drawbacks including poor bone tissue targeting, low bioavailability, and significant systemic side effects, which limit its therapeutic efficacy and application [4,5]. Therefore, the development of targeted delivery systems capable of precisely transporting therapeutic agents to bone lesions is crucial for achieving efficient and low-toxicity treatment.

Nanotechnology offers a revolutionary solution to this challenge. Among various nanocarriers, lipid nanoparticles (LNPs) are considered a highly promising drug delivery platform due to their excellent biocompatibility, biodegradability, and high drug-loading capacity [6,7]. Microfluidic technology, as an advanced platform for nanoparticle fabrication, enables precise control over the mixing of the lipid and aqueous phases, producing LNPs with uniform size, narrow size distribution, and excellent batch-to-batch reproducibility [8]. This technique allows for the fine-tuning of nanoparticle physicochemical properties by adjusting flow parameters such as total flow rate and flow rate ratio, making it particularly suitable for the development of functionalized delivery systems with well-defined particle characteristics [9]. The total flow rate is a key parameter that determines mixing efficiency and particle size. Notably, good size uniformity not only facilitates batch-to-batch consistency but also contributes to the homogeneous distribution of nanoparticles within bone tissue, thereby providing a foundation for subsequent targeted delivery.

A key strategy for achieving bone targeting involves modifying the LNP surface with bone-affinity targeting molecules [10]. Among numerous candidate ligands, the bisphosphonate drug alendronate sodium (Alen) exhibits extremely high affinity for hydroxyapatite (HAP), the main inorganic component of the bone matrix [11], and is itself widely used clinically as an anti-bone resorption agent [12], making it an ideal choice for constructing bone-targeted delivery systems. Previous studies have demonstrated that conjugation of Alen to nanoparticle surfaces effectively mediates specific binding to bone tissue, exhibiting favorable targeting efficacy across various delivery systems. For instance, Jing et al. modified Alen onto polymeric nanoparticle surfaces and demonstrated in osteoporotic mouse models that this modification significantly enhanced nanoparticle accumulation in bone tissue and effectively inhibited osteoclast activity [13]; similarly, Wu et al. conjugated Alen to liposome surfaces and achieved bone-targeted delivery of anti-tumor drugs in bone metastasis models, significantly suppressing tumor growth [14]. Although these studies indicate that Alen modification strategies have achieved promising results in conventional nanocarriers, how to achieve efficient, stable, and controllable modification of Alen onto the LNP surface remains a technical challenge that determines its targeting performance and druggability [15].

For the surface functionalization of LNPs with targeting ligands, two main strategies are commonly employed: the pre-modification method and the post-modification method. The pre-modification method involves synthesizing the ligand-lipid conjugate prior to nanoparticle assembly and directly incorporating it into the lipid mixture during formulation. In contrast, the post-modification method involves first preparing blank nanoparticles bearing reactive functional groups, followed by covalent conjugation of the targeting ligand to the pre-formed nanoparticle surface [16]. Both strategies have been widely used to construct targeted LNPs using various ligands, including peptides, antibodies, and small molecules. Examples of pre-modification include Sakurai et al., who incorporated cyclic RGD-PEG-DSPE into liposomes to prepare αvβ3 integrin-targeted delivery systems for anti-angiogenic therapy of renal cell carcinomas [17]; and Kasiewicz et al., who incorporated a multivalent GalNAc ligand (GL6) into LNPs to prepare asialoglycoprotein receptor-targeted delivery systems for delivering CRISPR base editors to the liver of patients with low LDLR activity [18]. The post-modification method has also shown promising potential. For example, Cohen et al. covalently conjugated hyaluronic acid (HA) to amine-functionalized LNPs via EDC/NHS chemistry to achieve targeted delivery to CD44-positive cells [19]; and Herrera-Barrera et al. conjugated the peptide MH42 to LNP surfaces via NHS-PEG-DSPE, enabling subretinal targeted delivery in a non-human primate model [20]. Although these studies demonstrate the effectiveness of both strategies in various targeting contexts, comparative studies on modifying Alen onto LNP surfaces for bone-targeted delivery remain limited.

To address this, the present study utilized microfluidic technology to construct Alen-modified lipid nanoparticles for bone-targeted mRNA delivery. First, the microfluidic process parameters were examined by investigating the effect of total flow rate on LNP size and uniformity, establishing suitable preparation conditions. On this basis, the pre-conjugation and post-conjugation strategies were compared for their application in Alen-modified LNPs, clarifying the influence of each strategy on Alen functionalization. In addition, the physicochemical properties, *in vitro* targeting capability, *in vivo* biodistribution, and biosafety of the constructed bone-targeted delivery system were evaluated, aiming to establish a robust and reproducible bone-targeted mRNA delivery platform to support the treatment of skeletal disorders.

## 2. Materials and Methods

### 2.1. Main Materials and Reagents

1,2-distearoyl-sn-glycero-3-phosphoethanolamine-polyethylene glycol 2000-N-hydroxysuccinimide ester (DSPE-PEG2000-NHS) was purchased from Shanghai Ponsure Biotechnology Co., Ltd. (Shanghai, China). Cholesterol, 1,2-dimyristoyl-rac-glycero-3-methoxypolyethylene glycol-2000 (DMG-PEG2000) and 1,2-distearoyl-sn-glycero-3-phosphocholine (DSPC) were purchased from Shanghai Aivite Medical Technology Co., Ltd. (Shanghai, China). (6Z,9Z,28Z,31Z)-heptatriaconta-6,9,28,31-tetraen-19-yl 4-(dimethylamino)butanoate (DLin-MC3-DMA) was purchased from Shanghai Seebio Biotech Co., Ltd. (Shanghai, China). Alendronate sodium (Alen) was purchased from Sigma-Aldrich (St. Louis, MO, USA). Phosphate-buffered saline (PBS, pH 7.4), absolute ethanol, and other analytical grade reagents were purchased from Sinopharm Chemical Reagent Co., Ltd. (Shanghai, China). Hydroxyapatite (HAP) nanopowder for *in vitro* targeting validation was purchased from Aladdin Biochemical Technology Co., Ltd. (Shanghai, China). Membrane dye DiD (1,1′-dioctadecyl-3,3,3′,3′-tetramethylindodicarbocyanine perchlorate) was purchased from Beyotime Biotechnology Co., Ltd. (Shanghai, China). Green fluorescent protein (GFP) mRNA was purchased from GenScript Biotech Corporation (Nanjing, China). Quant-iT RiboGreen RNA Assay Kit was purchased from Thermo Fisher Scientific (Waltham, MA, USA).

### 2.2. Experimental Animals

The protocol was approved by the Institutional Animal Care and Use Committee of Shenzhen University Medical School on 11 December 2023 (No. IACUC-202300185). Female C57BL/6J mice (aged 6–8 weeks) were obtained from Guangdong Medical Laboratory Animal Center (Guangzhou, China). All animal experiments were conducted in strict accordance with the relevant regulations of the Institutional Animal Care and Use Committee of Shenzhen University Medical School.

### 2.3. Synthesis and Purification of the Bone-Targeting Ligand DSPE-PEG-Alen

DSPE-PEG2000-NHS (5.0 mg) and Alen (10.0 mg) were accurately weighed and co-dissolved in 2 mL of phosphate-buffered saline (PBS, pH 8.0). The reaction mixture was stirred magnetically in the dark for 8 h to allow sufficient amidation coupling between the NHS active ester and the primary amine group of Alen. After the reaction, the mixture was transferred to a dialysis bag (molecular weight cutoff (MWCO) 3.5 kDa, Yuan Ye Biological Technology Co., Ltd., Shanghai, China) and dialyzed against ultrapure water at 4 °C for 24 h, with the dialysis solution changed 6 times, to thoroughly remove unreacted Alen and byproducts. Finally, the liquid inside the dialysis bag was lyophilized to obtain the white flocculent solid product DSPE-PEG-Alen, which was sealed and stored at −20 °C for future use. The chemical structure of the product was confirmed by proton nuclear magnetic resonance spectroscopy (^1^H NMR, AVANCE NEO 400 MHz, Bruker, Fällanden, Switzerland) and Fourier transform infrared spectroscopy (FT-IR, Nicolet iS50, Thermo Fisher Scientific, Waltham, MA, USA).

### 2.4. Preparation and Targeted Modification of Lipid Nanoparticles

#### 2.4.1. Microfluidic Preparation and Process Optimization of Lipid Nanoparticles

Blank lipid nanoparticles (LNPs) were first prepared using microfluidic technology (LSP01-3A, Baoding Lange Constant Flow Pump Co., Ltd., Baoding, China). A Y-shaped microfluidic chip (channel depth: 100 μm, channel width: 200 μm) was employed for all preparations. The organic phase consisted of ionizable lipid, cholesterol, DSPC, and DMG-PEG2000 dissolved in absolute ethanol at a molar ratio of 50:38.5:10:1.5, with a total lipid concentration of 10 mg/mL. The aqueous phase was 50 mM sodium citrate buffer (pH 4.0). To evaluate the effect of total flow rate, three different flow rates were compared at a fixed flow rate ratio between the two phases (organic phase:aqueous phase = 1:3, *v*/*v*): a low-speed group with a total flow rate of 4 mL/min (organic phase 1 mL/min, aqueous phase 3 mL/min), a medium-speed group with a total flow rate of 8 mL/min (organic phase 2 mL/min, aqueous phase 6 mL/min), and a high-speed group with a total flow rate of 12 mL/min (organic phase 3 mL/min, aqueous phase 9 mL/min). After mixing, the effluent was collected and dialyzed using dialysis bag (MWCO 8–14 kDa, Yuan Ye Biological Technology Co., Ltd., Shanghai, China) at 4 °C for 24 h to remove ethanol, with four changes of the dialysis buffer. The resulting LNP suspension was sterilized by filtration through a 0.22 μm filter to obtain blank LNPs. The particle size, polydispersity index (PDI), and Zeta potential of each group were characterized by dynamic light scattering (DLS, Zetasizer Nano ZS, Malvern Panalytical, Malvern, UK) to identify the optimal microfluidic parameters with the best comprehensive performance, which were subsequently used for the preparation of all functionalized nanoparticles.

Using the optimized microfluidic parameters (Y-shaped chip, total flow rate 8 mL/min, organic:aqueous = 1:3, *v*/*v*), two types of functionalized LNPs were prepared as follows:

(1) GFP-mRNA-loaded lipid nanoparticles: GFP-mRNA was added to the aqueous phase at a final concentration of 100 μg/mL, while the remaining composition was identical to that of blank LNPs. After mixing using microfluidic technology, the sample was dialyzed under the same conditions as blank LNPs (MWCO 8–14 kDa, protected from light at 4 °C for 24 h with four changes of dialysis buffer) to remove ethanol. Following dialysis, the suspension was sterilized by filtration through a 0.22 μm filter to obtain LNP@GFP-mRNA.
(2) DiD-labeled lipid nanoparticles: Fluorescent labeling was achieved by directly incorporating the membrane dye DiD into the organic phase, with a mass ratio of DiD to total lipids of 1:100. The remaining composition of the organic phase was identical to that of blank LNPs. The aqueous phase was prepared according to the experimental objective: for the preparation of empty DiD-labeled LNPs, pH 4.0, 50 mM sodium citrate buffer was used; for the preparation of DiD-labeled LNPs co-loaded with GFP-mRNA, the same buffer was supplemented with GFP-mRNA at a final concentration of 100 μg/mL. After mixing via microfluidic technology, the resulting suspension was diluted with 50 volumes of PBS and then concentrated back to the original volume by ultrafiltration using an ultrafiltration tube (MWCO 50 kDa, 4 °C) to remove ethanol and free DiD. Finally, the suspension was sterilized by filtration through a 0.22 μm filter to obtain DiD-labeled LNPs.

#### 2.4.2. Construction and Comparison of Alen Targeting Modification Strategies

Based on the optimized process described above, two Alen modification strategies were constructed and compared:

(1) Pre-conjugation method: The pre-synthesized targeting ligand DSPE-PEG-Alen was directly used as one of the lipid components. It was mixed with other lipids and then co-assembled in one step using the optimal microfluidic parameters to obtain Alen-modified LNPs (denoted as Pre-Alen-LNPs).
(2) Post-conjugation method: First, LNP precursors with reactive NHS esters on their surface (LNP-NHS) were prepared using the reactive lipid DSPE-PEG2000-NHS. Subsequently, LNP-NHS was reacted with excess Alen in pH 7.4 phosphate buffer at 4 °C for 8 h, allowing Alen to be covalently linked to the particle surface via amide bonds. After dialysis purification, Alen-modified LNPs were obtained (denoted as Post-Alen-LNPs).

All finally obtained nanoparticle suspensions were stored at 4 °C protected from light and used for subsequent characterization and evaluation experiments within 7 days.

### 2.5. Characterization of Nanoparticle Physicochemical Properties

#### 2.5.1. Transmission Electron Microscopy Analysis

The morphology and microstructure of Alen-LNP nanoparticles were observed using a transmission electron microscope (TEM, Hitachi HT7700, Tokyo, Japan). Briefly, the freshly prepared nanoparticle suspension was diluted with deionized water to an appropriate concentration. A 10 μL aliquot of the diluted sample was dropped onto a carbon film-coated copper grid and allowed to stand at room temperature for 3 min. Excess liquid was carefully absorbed from the edge using filter paper. Subsequently, 10 μL of a 2% (*w*/*v*) phosphotungstic acid solution was dropped onto the grid for negative staining for 1 min, after which the staining solution was again removed with filter paper. The sample was dried at room temperature. After complete drying, observation and image acquisition were performed at an accelerating voltage of 120 kV. The spherical morphology, uniformity, and approximate size of the nanoparticles were analyzed from the TEM images.

#### 2.5.2. Dynamic Light Scattering and Zeta Potential Analysis

The hydrated particle size distribution, polydispersity index (PDI), and Zeta potential of the nanoparticles were measured using a Malvern particle size analyzer (Zetasizer Nano ZS90, Malvern Panalytical, Malvern, UK). Particle size measurement was based on the principle of dynamic light scattering: the sample was diluted with deionized water to an appropriate concentration (aiming for a scattered light intensity count value within the instrument’s optimal range), injected into a disposable polystyrene cuvette, equilibrated at 25 °C for 2 min, and then measured. Zeta potential measurement was based on the principle of electrophoretic light scattering: the diluted sample was injected into a dedicated folded capillary electrophoresis cell, and the Zeta potential value was calculated using the instrument’s built-in Smoluchowski model at the same temperature.

#### 2.5.3. Encapsulation Efficiency Determination

The encapsulation efficiency (EE) of the lipid nanoparticles was determined using the Quant-iT RiboGreen RNA Assay Kit. Briefly, the RiboGreen working solution was diluted with TE buffer according to the manufacturer’s instructions, and a standard curve was established using mRNA standards of known concentrations. The LNP sample to be tested was divided into two aliquots: one aliquot was treated with 1% Triton X-100 solution for complete lysis to release all encapsulated mRNA, and its fluorescence intensity was measured as the total RNA amount (A_total_). The other aliquot was left untreated, and its fluorescence intensity was directly measured as the free RNA amount (A_free_). The fluorescence values of each well were read using a multifunctional microplate reader at excitation/emission wavelengths of 480/520 nm. Each sample was tested in triplicate. The encapsulation efficiency was calculated using the following formula:

(1) $$\mathrm{EE}\;(\%)\;=\;\frac{A_{\mathrm{total}}\;-\;A_{\mathrm{free}}}{A_{\mathrm{total}}}\;\times \;100$$

### 2.6. In Vitro Cell Experiments

#### 2.6.1. Isolation and Culture of Bone Marrow Mesenchymal Stem Cells (BMSCs)

Bone marrow mesenchymal stem cells were used for functional evaluation in this experiment [21]. Femurs and tibias were isolated from 6–8-week-old C57BL/6J mice. The bone marrow cavity was flushed with complete α-MEM medium containing 10% fetal bovine serum to collect the cell suspension. After centrifugation and resuspension, the cells were seeded into culture dishes. The cells were cultured in an incubator at 37 °C with 5% CO_2_, and the medium was changed every 3 days. When cells reached 80–90% confluence, they were digested with 0.25% trypsin and passaged. Cells from passages 3–5 were used for subsequent experiments.

#### 2.6.2. Cellular Uptake Experiment

To verify whether Alen-LNPs could be effectively taken up by BMSCs and achieve functional mRNA delivery, a qualitative analysis was performed using confocal microscopy. BMSCs in the logarithmic growth phase were seeded at an appropriate density in confocal dishes. After cell attachment, the medium was replaced with medium containing either bone-targeting LNPs loaded with mRNA encoding green fluorescent protein (Alen-LNP@GFP-mRNA) or unmodified LNPs loaded with GFP-encoding mRNA (LNP@GFP-mRNA). After 24 h of incubation, the medium was discarded, and the cells were gently washed three times with PBS and fixed with 4% paraformaldehyde at room temperature for 15 min. After fixation, the cells were washed again with PBS, and the nuclei were stained using an anti-fade mounting medium containing 4′,6-diamidino-2-phenylindole (DAPI) (1 μg/mL). After staining, the cells were immediately observed under a laser scanning confocal microscope (LSM 880, Carl Zeiss, Oberkochen, Germany). The cellular uptake of nanoparticles and their mediated mRNA translation efficiency were analyzed by observing the fluorescence signals [22].

#### 2.6.3. Cytotoxicity Assay (CCK-8 Method)

To assess the cytotoxicity of Alen-LNP on BMSCs, cell viability was detected using the Cell Counting Kit-8 (CCK-8) method. BMSCs were seeded in a 96-well plate at a density of 5 × 10^3^ cells per well and cultured for 24 h to allow attachment. Different concentrations of Alen-LNP were set (0, 8, 16, 25, 50, 100, 200 μg/mL lipid concentration). Each group had three replicate wells. After treatment with the nanoparticle-containing medium for 48 h, fresh medium containing 10% CCK-8 reagent was added to each well, and incubation continued for 1 h. The absorbance of each well was measured at 450 nm using a microplate reader (Multiskan SkyHigh, Thermo Fisher Scientific, Waltham, MA, USA). Cell viability was calculated using the following formula:

(2) $$\mathrm{Cell}\;\mathrm{viability}\;(\%)\;=\;\frac{{\mathrm{OD}}_{\mathrm{experiment}}\;-\;{\mathrm{OD}}_{\mathrm{blank}}}{{\mathrm{OD}}_{\mathrm{control}}\;-\;{\mathrm{OD}}_{\mathrm{blank}}}\;\times \;100$$

where OD_experiment_ is the optical density of cells treated with the tested agents, OD_control_ is the optical density of untreated control cells, and OD_blank_ is the optical density of the background.

### 2.7. In Vitro Bone-Targeting Ability Evaluation

#### 2.7.1. Hydroxyapatite (HAP) Binding Assay

HAP powder (10 mg) was precisely weighed and placed in a centrifuge tube. Then, 1 mL of DiD-labeled Alen-LNP or blank LNP suspension diluted with PBS (at lipid concentrations of 0.5 mg/mL and 1.0 mg/mL) was added. After incubation with shaking at 37 °C for 1 h, the mixture was centrifuged at 5000 rpm for 5 min. A 200 μL aliquot of the supernatant was carefully aspirated and transferred to a black 96-well plate. The absorbance of the supernatant was measured using a microplate reader (BioTek, Winooski, VT, USA) at an excitation wavelength of 640 nm. The binding rate was calculated using the formula:

(3) $$\mathrm{Binding}\;\mathrm{rate}\;(\%)\;=\;(1\;-\;\frac{\mathrm{Absorbance}\;\mathrm{after}\;\mathrm{HAP}\;\mathrm{incubation}}{\mathrm{Absorbance}\;\mathrm{before}\;\mathrm{HAP}\;\mathrm{incubation}})\;\times \;100$$

#### 2.7.2. Quantification of Alen on LNP Surfaces

Alen contains bisphosphonate groups that specifically chelate Cu^2+^ to form a colored complex, enabling quantification of Alen by absorbance measurement. A standard curve was first established by incubating Alen standards at concentrations of 1.25, 2.5, 5.0, 7.5, and 10.0 μg/mL with copper sulfate solution (10 mmol/L) for 30 min. The absorbance was measured using the same microplate reader at a characteristic wavelength (e.g., 270 nm) to generate a concentration-absorbance standard curve. Pre-Alen-LNP and Post-Alen-LNP samples were normalized to equal lipid concentrations, incubated with copper sulfate under the same conditions, and their absorbance was measured. The surface density of Alen ligands on LNPs prepared by the two conjugation strategies was then calculated based on the standard curve and compared.

#### 2.7.3. Bone Fragment Binding Assay

Mouse femurs were excised and carefully cleaned of adhering soft tissues. The bones were then fragmented into small pieces of approximately 1–2 mm^3^. The bone fragments were incubated with DiD-labeled nanoparticle suspension (diluted in PBS to the indicated lipid concentrations) for 2 h at room temperature under gentle shaking in the dark. After incubation, the supernatant was removed, and the bone fragments were washed thoroughly with PBS (three to five times) to eliminate unbound nanoparticles. The washed fragments were subsequently placed in confocal dishes and examined using a laser scanning confocal microscope to evaluate specific fluorescence binding on the bone surface.

### 2.8. In Vivo Experiments

#### 2.8.1. Evaluation of *In Vivo* Bone-Targeting Distribution

To evaluate the real-time *in vivo* distribution and bone-targeting characteristics of Alen-modified lipid nanoparticles (Alen-LNP), small animal *in vivo* fluorescence imaging was first performed. C57BL/6J mice were intravenously injected via the tail vein with DiD-labeled Alen-LNP or unmodified blank LNP. At 24 h post-injection, mice were anesthetized with isoflurane gas and placed in the dark chamber of a small animal *in vivo* optical imaging system (IVIS^®^ Spectrum, PerkinElmer, Waltham, MA, USA). Fluorescence images of the ventral side of the mice were acquired with excitation and emission wavelengths set at 640 nm and 680 nm, respectively. Imaging focused on the fluorescence signal intensity in limb joints (such as knees and ankles) and the spine region to preliminarily observe the enrichment of nanoparticles in bones.

After *in vivo* imaging, the mice were euthanized and immediately dissected. Major organs (heart, liver, spleen, lungs, kidneys) and complete femurs and tibias were collected sequentially. All isolated tissues were gently rinsed with PBS, and surface liquid was blotted dry with filter paper. The tissues were then arranged on a black background Petri dish for fluorescence imaging of all isolated organs and bones. After image acquisition, regions of interest (ROIs) were drawn on bone tissue and each organ using the instrument’s accompanying analysis software (Living Image software, version 4.5.5, PerkinElmer, Waltham, MA, USA) to quantitatively calculate their average fluorescence intensity. By comparing the fluorescence intensity ratio between bone tissue in the Alen-LNP group and the blank LNP group, the bone-targeting efficiency was assessed.

#### 2.8.2. *In Vivo* Bone Targeting and Cellular Delivery Verification Experiment

To verify the bone-targeting ability of Alen-LNP and its functional delivery to cells within bone tissue at the living organism level, bone tissue section analysis was conducted. C57BL/6J mice were intravenously injected via the tail vein with DiD-labeled Alen-LNP loaded with GFP-mRNA (DiD-Alen-LNP@GFP-mRNA). At 24 h post-injection, mice were euthanized, and femurs and tibias were isolated and cleaned of soft tissue. Bone tissue samples were fixed in 4% paraformaldehyde for 48 h, followed by decalcification in 14% ethylenediaminetetraacetic acid (EDTA) decalcification solution at 4 °C for 2–3 weeks, with regular changes of the solution. After complete decalcification, tissues were dehydrated through a graded ethanol series, embedded in paraffin, and sectioned consecutively at 5 μm thickness along the sagittal plane of the long bone axis. Finally, sections were mounted with an anti-fade mounting medium containing DAPI. Bone tissue sections were observed using a laser scanning confocal microscope. Through three-channel imaging (DAPI for nuclei, Alexa Fluor 488/GFP for protein expression, DiD for nanoparticles), the co-localization of DiD and GFP signals was analyzed to confirm nanoparticle cellular uptake and mRNA translation.

### 2.9. In Vivo Biosafety Evaluation

To evaluate the *in vivo* safety of Alen-LNP, histopathological analysis was performed. Healthy C57BL/6J mice were randomly divided into a PBS group and an Alen-LNP group. On day 7 after a single administration via the tail vein, mice were euthanized and rapidly dissected to completely remove the heart, liver, spleen, lungs, and kidneys. Tissues were rinsed with PBS and immediately immersed in 4% paraformaldehyde for fixation for 48 h. Subsequently, routine paraffin embedding was performed. Sections of 5 μm thickness were cut along the largest surface of each organ. After deparaffinization and rehydration, sections were stained with hematoxylin and eosin (H&E) and observed under an optical microscope. The focus was on assessing the integrity of tissue structure and the presence of pathological changes such as inflammatory cell infiltration, edema, degeneration, or necrosis.

### 2.10. Statistical Analysis

All data were derived from at least three independent experiments (*n* denoted the number of independent experiments; *n* = 3) and were presented as means. One-way analysis of variance (ANOVA) followed by Tukey’s test was used for multi-group comparisons, while Student’s *t*-test was used for two-group comparisons. A *p*-value < 0.05 was considered statistically significant. Statistical analyses were performed using GraphPad Prism 9.0 (GraphPad Software, San Diego, CA, USA) and Origin 2024 (OriginLab, Northampton, MA, USA).

## 3. Results

### 3.1. Microfluidic Preparation Process Optimization and Nanoparticle Physicochemical Properties

**Figure 1:**
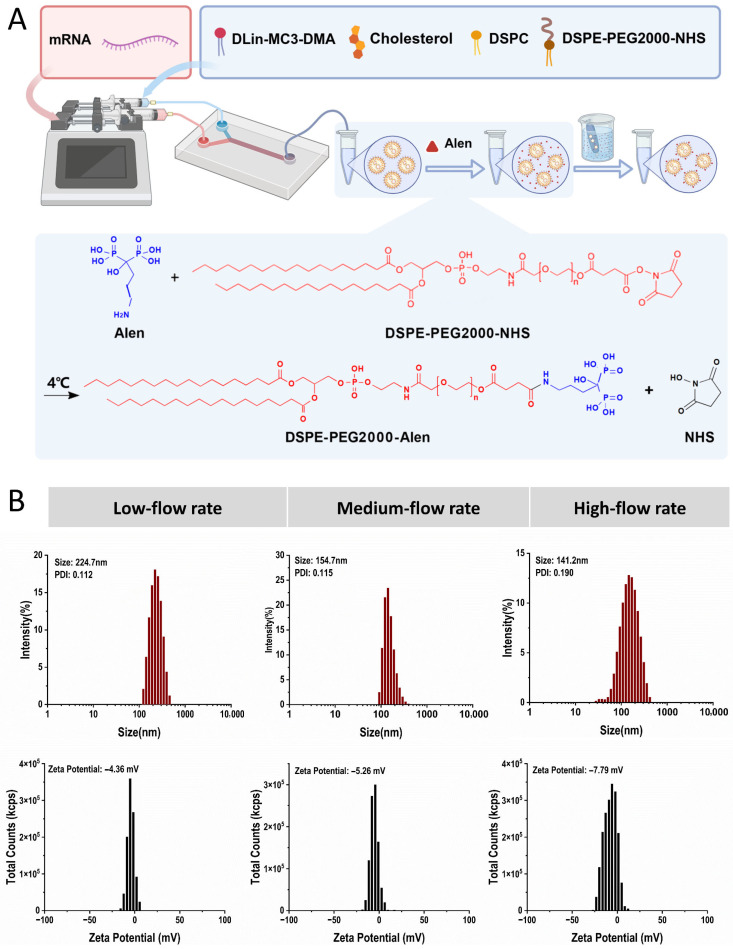
Optimization of the microfluidic preparation process for lipid nanoparticles (LNPs). (**A**) Schematic illustration of the microfluidic-based LNP fabrication process, depicting the controlled mixing of lipid (organic) and buffer (aqueous) phases within the chip to drive nanoprecipitation and self-assembly. (**B**) Particle size, polydispersity index (PDI), and Zeta potential of blank LNPs prepared at different total flow rates (Low: 4 mL/min; Medium: 8 mL/min; High: 12 mL/min).

To obtain lipid nanoparticles with uniform particle size and good stability, microfluidic technology was selected for preparation. Microfluidics enables precise control over the mixing process of two-phase fluids, allowing active regulation of nanoparticle nucleation and growth kinetics, thereby significantly improving particle size uniformity and batch-to-batch reproducibility compared to traditional passive assembly methods [23]. Briefly, the preparation process involved dissolving lipids in ethanol as the organic phase and an acidic buffer as the aqueous phase (Figure 1A). These were precisely infused via syringe pumps, converged at high speed within the specific structure of the microfluidic chip, and uniformly mixed utilizing chaotic advection to promote lipid molecular self-assembly and formation of encapsulated nanoparticles. Following preparation, the obtained LNPs bearing NHS-reactive groups were incubated with Alen at 4 °C to allow conjugation between DSPE-PEG-NHS and the amino groups of Alen, forming DSPE-PEG-Alen on the nanoparticle surface. This post-conjugation method enabled covalent conjugation of Alen onto the pre-formed LNP surface. The resulting mixture was subsequently dialyzed to remove unconjugated Alen and byproducts, yielding purified bone-targeted Alen-LNPs. Among the parameters, the total flow rate is a key factor determining the shear force and Reynolds number within the mixing chamber, directly affecting lipid molecular diffusion and self-assembly efficiency, and consequently determining the final particle size and distribution [24,25]. Therefore, investigating the impact of different total flow rates is crucial for particle size and uniformity.

Under the condition of a fixed organic phase (ethanol) to aqueous phase (buffer) flow rate ratio of 1:3, three groups of total flow rates—low (4 mL/min), medium (8 mL/min), and high (12 mL/min)—were compared. Dynamic light scattering characterization results (Figure 1B) showed that the total flow rate significantly affected the physicochemical properties of the LNPs. In the low-speed group, weaker shear forces might lead to insufficient lipid mixing and incomplete dispersion of aggregates, resulting in larger particle sizes. In the high-speed group, excessively strong shear forces might interfere with the ordered self-assembly process of lipid molecules, easily generating aggregates with uneven, small particle sizes, with a polydispersity index (PDI) greater than 0.15, indicating a wider distribution. In contrast, the medium-speed group achieved an optimal balance in fluid dynamics. The blank LNPs prepared under this condition exhibited the best comprehensive performance: a hydrated hydrodynamic diameter of 154.7 nm, a PDI of 0.115, and a Zeta potential of −5.26 mV. This size is suitable for *in vivo* delivery, and the narrow particle size distribution and moderate surface charge are beneficial for system stability [26]. Therefore, this optimized medium-speed parameter was adopted for the preparation of all subsequent Alen-functionalized LNPs to ensure that functionalization modifications were based on high-quality nanoparticles.

### 3.2. Physicochemical Characterization of Alen-LNPs

**Figure 2:**
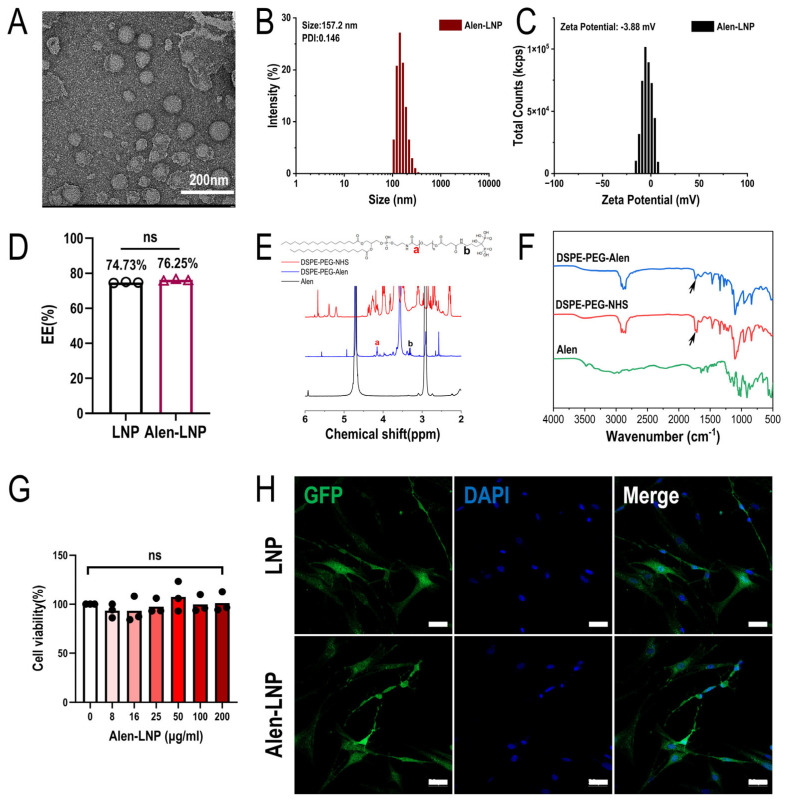
Physicochemical and structural characterization of Alen-modified lipid nanoparticles. (**A**) Representative TEM image of Alen-LNPs showing spherical morphology and uniform dispersion (Scale bar: 200 nm). (**B**) Particle size distribution of Alen-LNPs measured by DLS. (**C**) Zeta potential distribution of Alen-LNPs. (**D**) Comparison of mRNA encapsulation efficiency between Alen-LNPs and unmodified LNPs (*n* = 3). (**E**) ^1^H NMR spectra of DSPE-PEG-NHS and DSPE-PEG-Alen. The letters “a” and “b” in panel (**E**) indicate the common characteristic peak of the DSPE-PEG backbone and DSPE-PEG-Alen, respectively, as described in the main text. The appearance of new characteristic peaks in the product spectrum confirms successful Alen conjugation. (**F**) FT-IR spectra of DSPE-PEG-NHS and DSPE-PEG-Alen. The attenuation of NHS ester carbonyl peaks (1810 and 1780 cm^−1^, indicated by black arrows) verifies amide bond formation with Alen. (**G**) Cytotoxicity of Alen-LNPs against BMSCs assessed by CCK-8 assay after 48 h incubation (*n* = 3). Black circles represent individual values from replicate samples. (**H**) Confocal microscopy images showing cellular uptake of GFP mRNA-loaded Alen-LNPs and unmodified LNPs by BMSCs and subsequent GFP expression (green). Nuclei were stained with DAPI (blue) (Scale bar: 50 μm). In panels (**D**,**G**), “ns” indicates no significant difference (*p* > 0.05).

Using the optimal microfluidic preparation process, Alen-modified LNPs were successfully fabricated. Transmission electron microscopy observation (Figure 2A) showed that the prepared nanoparticles had a regular morphology, appearing as uniformly dispersed spheres or near-spheres, indicating a stable microfluidic mixing process and good lipid self-assembly. Dynamic light scattering analysis results indicated that the Alen-LNPs had an average hydrated hydrodynamic diameter of 157.2 nm (Figure 2B), a polydispersity index (PDI) of 0.146, and a Zeta potential of −3.88 mV (Figure 2C), suggesting a relatively uniform particle size distribution. Compared to blank LNPs, the particle size and zeta potential of the modified nanoparticles exhibited no significant changes, while the size distribution remained within a narrow range. To evaluate whether Alen modification affected the drug-loading performance of the LNPs, the encapsulation efficiency was further determined. Figure 2D showed that the encapsulation efficiency of Alen-LNPs was 76.25%, showing no significant difference compared to the 74.73% of blank LNPs (*p* > 0.05). These results indicate that the Alen-LNPs prepared through the optimized process possess favorable physicochemical properties and stable drug-loading performance, laying a foundation for subsequent biological function studies [27].

### 3.3. Synthesis and Characterization of the Bone-Targeting Ligand DSPE-PEG-Alen

The Alen molecule contains two phosphate groups that can form stable chelates with Ca^2+^ in bone hydroxyapatite (HAP), conferring high bone affinity. Therefore, the successful modification of the bone-targeting ligand is a key factor determining the targeting efficiency of lipid nanoparticles (LNPs). This study systematically compared two modification strategies: “pre-conjugation” and “post-conjugation”. The “pre-conjugation” strategy requires the pre-covalent linkage of the targeting molecule Alen to the lipid material to synthesize the DSPE-PEG-Alen conjugate, which is then used in LNP assembly. Thus, the synthesis and structural confirmation of this key intermediate are the first steps in evaluating the feasibility of the “pre-conjugation” method and ensuring that subsequent nanoparticles possess the expected targeting function [28].

The successful synthesis of DSPE-PEG-Alen was fundamental. The product was characterized by FT-IR and ^1^H NMR. ^1^H NMR spectral analysis (Figure 2E) showed that the characteristic peak (signal a) at a chemical shift (δ) of 4.1–4.3 ppm corresponded to the -HN-CO-CH_2_-O-methylene group in the DSPE-PEG backbone. This peak was common to both DSPE-PEG-NHS and DSPE-PEG-Alen; therefore, its integration represented the total amount of DSPE-PEG (including both unconjugated and conjugated forms). Simultaneously, a new characteristic peak (signal b) appeared in the range of δ 3.25–3.45 ppm, corresponding to the methylene protons in the -CO-NH-CH_2_-Alen structure formed after Alen conjugation; its integration represented only the content of the product DSPE-PEG-Alen. The synthesis efficiency, calculated from the integration ratio of signal b to signal a, was 93%. The FT-IR spectrum (Figure 2F) provided further verification: in the spectrum of DSPE-PEG-Alen, the characteristic carbonyl absorption peaks of the NHS ester at approximately 1810 cm^−1^ and 1780 cm^−1^ were significantly weakened. These data fully demonstrate that Alen was successfully covalently linked to the terminus of DSPE-PEG via an amide bond, efficiently synthesizing the desired targeting ligand.

### 3.4. In Vitro Cellular Uptake and Cytotoxicity

#### 3.4.1. Cytotoxicity of Alen-LNPs

To assess the *in vitro* biosafety of Alen-modified lipid nanoparticles (Alen-LNP), a CCK-8 cytotoxicity assay was performed. To determine an appropriate concentration range for testing, reference was made to systematic cytotoxicity data for LNP-siRNA reported in the literature, which indicated no toxicity up to 16 μg/mL in adherent A549 cells and 128 μg/mL in suspension HL60 cells [29]. Based on this safety window and considering potential working concentrations in subsequent application scenarios, the upper limit was extended to 200 μg/mL to explore a broader concentration range, and gradient concentrations from 0 to 200 μg/mL were evaluated. The results (Figure 2G) showed that after co-culturing Alen-LNP with BMSCs for 48 h, cell viability remained above 95% across all tested concentrations, with no significant difference compared to the PBS control group. These results demonstrate that the prepared nanoparticles possess good *in vitro* biocompatibility [30].

#### 3.4.2. Cellular Uptake and mRNA Translation

Within this safe concentration range, the functional delivery capability of the nanoparticles was further verified. BMSCs were co-incubated with either Alen-LNP@GFP-mRNA or LNP@GFP-mRNA for 24 h. Laser scanning confocal microscopy observation (Figure 2H) revealed obvious green fluorescence signals within the cells in both groups, indicating successful GFP protein expression. Visual inspection suggested no apparent difference in cellular uptake and mRNA translation efficiency between the Alen-LNP and unmodified LNP groups. This result demonstrated that after Alen modification, the LNPs could still be effectively internalized by BMSCs and successfully deliver functional mRNA, which was then translated into protein. These findings preliminarily confirmed their potential as a nucleic acid drug delivery vector [31].

### 3.5. In Vitro Bone-Targeting Performance

#### 3.5.1. HAP Binding Affinity

Hydroxyapatite (HAP) is the main inorganic component of the bone matrix, and Alen has a specific high affinity for it. Therefore, the HAP binding assay can directly and quantitatively evaluate the *in vitro* bone-targeting ability of nanoparticles [32]. This experiment was conducted to compare the effectiveness of the “pre-conjugation” and “post-conjugation” modification strategies at the physicochemical level, as the modification method may directly affect the orientation, density, and HAP binding accessibility of the Alen ligand on the nanoparticle surface [33].

**Figure 3:**
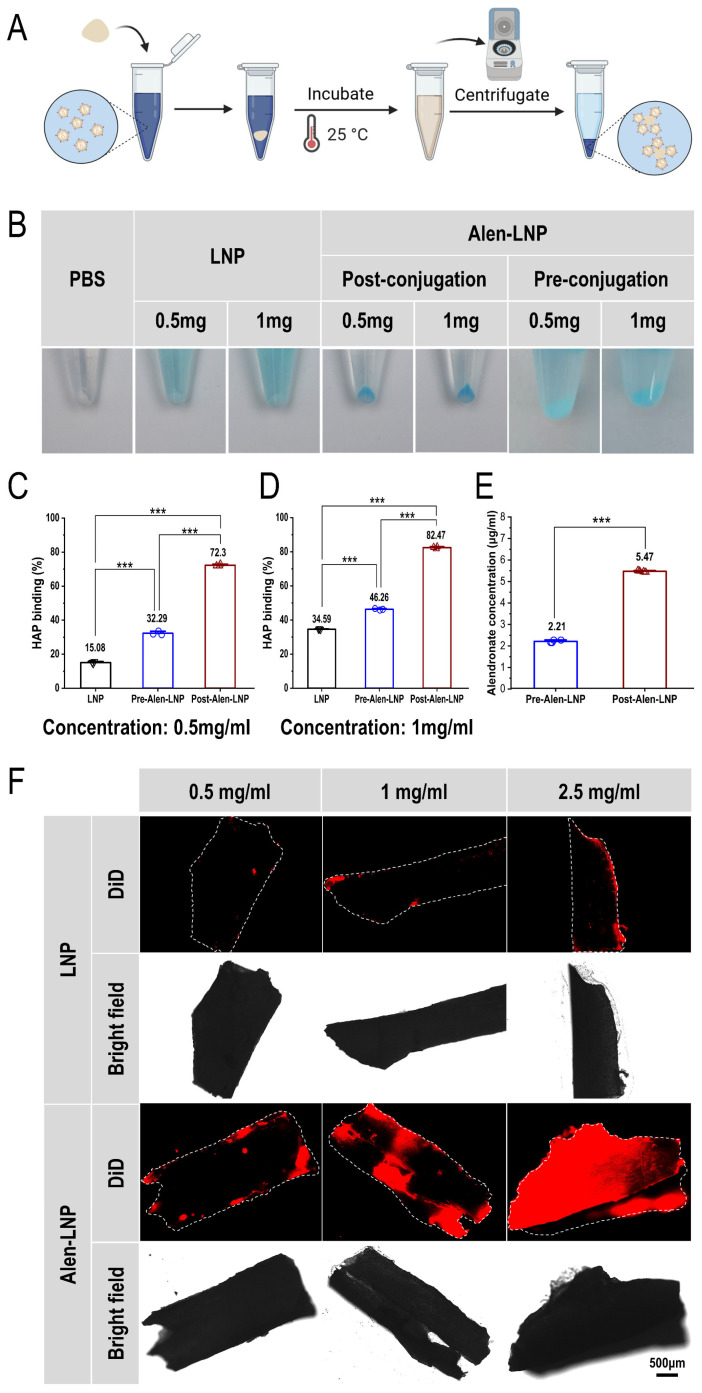
Comparison of *in vitro* bone-targeting capabilities of Alen-LNPs prepared by pre-conjugation and post-conjugation methods. (**A**) Schematic illustration of the hydroxyapatite (HAP) binding assay procedure. (**B**) Representative photographs of pelleted samples after incubation with HAP, including PBS control, unmodified LNPs, Pre-Alen-LNP and Post-Alen-LNP. The darkest coloration observed in the Post-Alen-LNP pellet indicates superior binding affinity. (**C**,**D**) Quantitative analysis of HAP binding rates for each group at lipid concentrations of 0.5 mg/mL (**C**) and 1.0 mg/mL (**D**) (*n* = 3). The post-conjugation method yields significantly higher binding affinity compared to the pre-conjugation approach. (**E**) Comparison of Alen mass concentration on Pre-Alen-LNP and Post-Alen-LNP surfaces (*n* = 3). (**F**) Representative confocal fluorescence images of mouse femoral bone fragments incubated with DiD-labeled Alen-LNPs or unmodified LNPs at varying concentrations. Red fluorescence represents DiD-labeled LNPs, dashed lines outline bone fragments, and black regions in bright-field images represent bone fragments. Alen-LNPs exhibit concentration-dependent and specific binding to the bone surface, while only minimal background fluorescence is detected in the LNP control group. Scale bar: 500 μm.*** *p* < 0.001 indicates a statistically significant difference for panels (**C**–**E**).

First, after incubation with HAP, the DiD-labeled post-conjugation group (Post-Alen-LNP) (Figure 3A) showed the deepest blue color in the centrifuged pellet, and the color was similarly deep at both 0.5 and 1.0 mg/mL concentrations, suggesting strong binding ability with non-significant concentration dependence. In contrast, the pellets from the pre-conjugation group (Pre-Alen-LNP) and the blank LNP group were much lighter in color (Figure 3B), indicating weaker affinity for HAP. This observation intuitively suggested that the post-conjugation strategy might be more favorable for Alen to exert its targeting binding function.

To quantitatively evaluate the binding efficiency, the absorbance of the supernatant was measured and the binding rate was calculated. The quantitative results were highly consistent with the qualitative observation: at a lipid concentration of 0.5 mg/mL, the HAP binding rates for Pre-Alen-LNP and Post-Alen-LNP were 32.29% and 72.3%, respectively (Figure 3C); at a concentration of 1.0 mg/mL, the binding rates increased to 46.26% and 82.47%, respectively (Figure 3D). These data indicate that Alen-LNPs prepared by both modification strategies exhibit affinity for HAP, although those prepared via the post-conjugation method demonstrated significantly higher HAP binding efficiency across the tested concentration range (*p* < 0.001). This difference can be attributed to the distinct ligand presentation modes of the two modification strategies. In the pre-conjugation method, Alen ligands are susceptible to steric hindrance or phase separation, resulting in insufficient surface exposure. In contrast, the post-conjugation method facilitates the oriented and high-density presentation of Alen ligands on the nanoparticle surface, thereby maximizing their binding capacity to HAP [34].

#### 3.5.2. Alen Density on LNP Surfaces

The mass concentration of Alen bound to Pre-Alen-LNP and Post-Alen-LNP surfaces was determined using the copper sulfate colorimetric method (Figure 3E). The results showed that the Alen concentration was 2.21 μg/mL for Pre-Alen-LNP and 5.47 μg/mL for Post-Alen-LNP, with the latter being significantly higher (*p* < 0.001). This result indicates that the post-conjugation strategy achieves greater Alen ligand loading on the LNP surface compared to the pre-conjugation approach.

#### 3.5.3. *In Vitro* Bone Fragment Binding Results

To validate the targeting performance of Alen-LNP prepared by the post-conjugation method in a three-dimensional model closer to the real complex microenvironment of bone tissue, a bone fragment *in vitro* incubation experiment was further conducted. DiD-labeled Alen-LNP and unmodified LNP at different lipid concentrations (0.5, 1.0, 2.5 mg/mL) were co-incubated with mouse femoral bone fragments, respectively.

Laser scanning confocal microscopy observation (Figure 3F) showed that at all three concentrations, the bone fragment surfaces in the Alen-LNP treatment group were attached with intense and specific red fluorescence signals. The fluorescence intensity increased with increasing nanoparticle concentration, showing a certain concentration dependence. In contrast, only extremely weak background fluorescence was detected on the bone fragment surfaces in the blank LNP group. This result visually confirms that the Alen ligand successfully modified via the post-conjugation method can effectively mediate the specific binding of nanoparticles to real bone matrix components (including organic and inorganic minerals), further supporting its good bone-targeting potential even in complex physiological environments.

### 3.6. In Vivo Bone-Targeting Distribution and Functional Delivery Verification

#### 3.6.1. *In Vivo* Bone-Targeting Distribution Results

**Figure 4:**
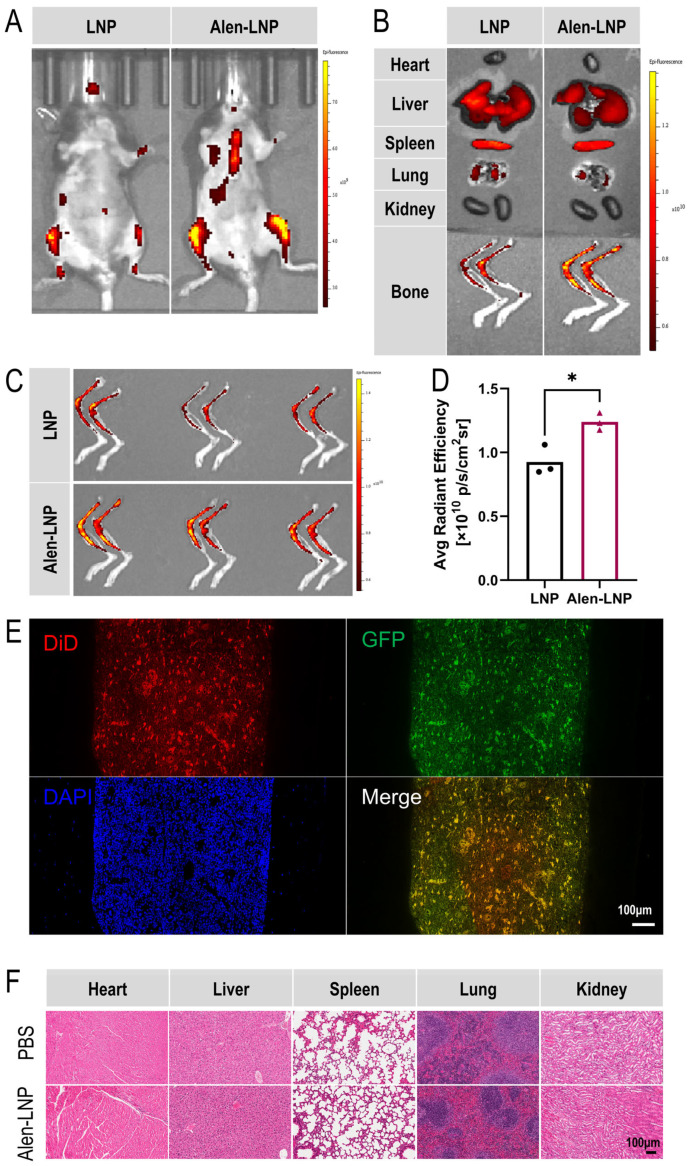
*In vivo* biodistribution, bone-targeted delivery, and biosafety evaluation of Alen-LNPs. (**A**) Representative *in vivo* fluorescence images of mice 24 h after intravenous injection of DiD-labeled Alen-LNPs or unmodified LNPs. (**B**) Ex vivo fluorescence images of major organs (heart, liver, spleen, lungs, kidneys) and hindlimb bones (femur, tibia) harvested from the mice shown in (**A**). (**C**) Ex vivo fluorescence images of femurs and tibias from mice (*n* = 3 per group) 24 h post-injection of DiD-labeled Alen-LNPs or unmodified LNPs. (**D**) Quantitative analysis of fluorescence intensity in bone tissues from (**C**). Data are presented as individual values (black and purple bullets; *n* = 3 per group). * *p* < 0.05 indicates a statistically significant difference. The results demonstrate significantly enhanced accumulation of Alen-LNPs compared to unmodified LNPs. (**E**) Fluorescence imaging of decalcified femur sections 24 h after injection of DiD-Alen-LNPs loaded with GFP mRNA. Blue: DAPI-stained nuclei; red: DiD-labeled nanoparticles; green: GFP reporter protein. Co-localization indicates successful nanoparticle uptake and mRNA translation. Scale bar: 100 μm. (**F**) H&E-stained sections of major organs harvested on day 7 post-administration, showing normal tissue morphology with no apparent pathological changes. Scale bar: 100 μm.

To evaluate the distribution characteristics of Alen-LNP prepared by the post-conjugation method in living animals, small animal *in vivo* and ex vivo fluorescence imaging experiments were conducted. Mice were intravenously injected with DiD-labeled Alen-LNP via the tail vein, and whole-body *in vivo* imaging was performed 24 h later. The results (Figure 4A) showed that compared to the control group injected with blank LNP, mice in the Alen-LNP group exhibited significant fluorescence enrichment signals in the limb joints and spine regions. For accurate quantification, mice were euthanized, and major organs (heart, liver, spleen, lungs, kidneys) and lower limb bones (femurs, tibias) were isolated for ex vivo imaging. Analysis revealed (Figure 4B) that the fluorescence intensity of Alen-LNP in major organs was comparable to that of the blank LNP group, indicating no significant difference in non-specific accumulation. Quantitative analysis of the region of interest (ROI) fluorescence intensity on the images of femurs and tibias from three parallel experiments (Figure 4C) showed that the average fluorescence intensity of Alen-LNP in bone tissue was 1.26 times that of the blank LNP group (Figure 4D), a difference that was statistically significant (*p* < 0.05). This quantitative result confirms at the *in vivo* level that Alen modification can effectively promote the specific enrichment of nanoparticles in bone tissue.

#### 3.6.2. *In Vivo* Functional mRNA Delivery Verification

The aforementioned *in vivo* imaging results confirmed the bone-targeting capability of Alen-LNP at the tissue level. However, whether nanoparticles enriched in bone tissue can be effectively internalized by target cells and subsequently accomplish functional gene delivery is key to assessing their therapeutic potential [35]. Therefore, this study further designed an experiment aimed at directly verifying whether this delivery system can achieve the complete functional chain from “tissue targeting” to “cellular uptake” to “protein expression” *in vivo*. Mice were intravenously injected via the tail vein with DiD-Alen-LNP@GFP-mRNA. At 24 h post-injection, femurs were harvested, and decalcified paraffin sections were prepared and subjected to DAPI staining, followed by observation using laser scanning confocal microscopy. The results (Figure 4E) showed clear co-localization of DiD red fluorescence (nanoparticles) and GFP green fluorescence (mRNA translation product) within cells in the bone marrow cavity. This result directly confirms: first, Alen-LNP enriched in bone tissue can be effectively internalized by relevant cells within the bone tissue; second, after internalization, the mRNA it carries can be successfully translated into functional protein intracellularly. This comprehensively demonstrates that the Alen-LNP delivery system not only possesses favorable bone-targeting properties but also has the potential to serve as a targeted gene therapy platform for bone diseases, capable of achieving precise delivery and functional expression of genetic drugs within target cells.

### 3.7. In Vivo Biosafety Results

To systematically evaluate the *in vivo* safety of Alen-LNP prepared by the post-conjugation method, histopathological analysis of major organs in mice was performed on day 7 post-administration (Figure 4F). Heart tissue sections showed that myocardial fibers were arranged neatly and consistently, with normal nuclear morphology and no congestion, edema, or inflammatory cell infiltration in the interstitium. In liver tissue, the lobular structure was clear and intact, hepatocytes appeared plump with uniform cytoplasm, and no inflammatory cell aggregation was observed. The spleen showed normal splenic corpuscle morphology without congestion or hemorrhage. Lung tissue exhibited intact alveolar structure with clear alveolar spaces and no inflammatory exudate in the interstitium. The kidneys showed clear demarcation between cortex and medulla, and glomerular morphology and structure were normal. Overall, in both the PBS control group and the Alen-LNP group, the tissue structures of all examined organs remained intact, cell morphology was normal, and no obvious pathological changes such as inflammatory cell infiltration, edema, cytoplasmic vacuolation, degeneration, or necrosis were observed. These results preliminarily indicate that, within the dose and observation period set in this experiment, Alen-LNP did not cause obvious acute systemic toxicity or organ damage, demonstrating good biosafety [36,37].

## 4. Discussion

This study aimed to investigate the effect of the total flow rate in microfluidic preparation on LNP fabrication, and to compare the impact of different modification strategies of Alen on the performance of bone-targeting lipid nanoparticles (LNPs). Furthermore, the physicochemical properties, targeting performance, and biosafety of the constructed system after targeted modification were evaluated. The main findings and discussions are as follows:

In terms of microfluidic process parameters, this study demonstrates that under a fixed two-phase flow rate ratio, the total flow rate is a critical parameter determining the size uniformity of LNPs. When the total flow rate was too low, insufficient fluid shear force led to inadequate mixing of lipid molecules, resulting in the formation of larger aggregates. Conversely, when the total flow rate was too high, excessive shear forces may interfere with the ordered self-assembly process of lipid molecules, causing uneven particle size distribution. In contrast, the medium flow rate condition (8 mL/min) achieved a better balance between fluid shear force and lipid molecular self-assembly kinetics. The LNPs prepared under this condition exhibited a particle size of approximately 154.7 nm with uniform distribution (PDI ~0.115), providing an ideal carrier foundation for subsequent functionalization modifications [38].

Based on the optimized process, Alen-modified LNPs were successfully prepared. Physicochemical characterization confirmed that the resulting Alen-LNPs exhibited regular morphology and good dispersibility, with no significant impact of Alen modification on particle size or zeta potential. In addition, the encapsulation efficiency of LNPs did not change significantly before and after modification, and the prepared Alen-LNPs maintained a good encapsulation efficiency. The CCK-8 assay results further supported the biocompatibility of Alen-LNPs, showing no obvious cytotoxicity toward BMSCs even at the highest tested concentration. The cellular uptake experiment further demonstrated that Alen modification did not compromise the ability of LNPs to deliver functional mRNA to BMSCs. Collectively, the Alen-LNPs prepared via the optimized process possessed favorable physicochemical properties and stable drug-loading performance, laying a solid foundation for subsequent biological function studies [39].

In the comparison of ligand synthesis and modification strategies, this study systematically investigated two strategies: the “pre-conjugation strategy” and the “post-conjugation strategy”. The pre-conjugation strategy involved mixing the DSPE-PEG-ligand with other lipid components prior to LNP formation, followed by co-assembly to form targeted LNPs. To this end, the DSPE-PEG-Alen ligand was first synthesized, and its successful synthesis was confirmed by nuclear magnetic resonance and Fourier-transform infrared spectroscopy, with a synthesis efficiency as high as 93%. In contrast, the post-modification method involved covalent coupling of the Alen ligand via chemical reaction onto pre-formed LNPs bearing reactive groups on their surface [40]. Through the above two modification strategies, Pre-Alen-LNPs and Post-Alen-LNPs were successfully constructed.

Alen has high affinity for hydroxyapatite in the bone matrix, and its modification strategy affects bone-targeting efficiency [41]. *In vitro* hydroxyapatite (HAP) binding assay results demonstrated that Alen-LNPs prepared by both modification strategies exhibited bone-targeting affinity. At a lipid concentration of 1.0 mg/mL, the pre-conjugation method achieved a binding rate of 46.26%, whereas the post-conjugation method achieved a binding rate of 82.47%, indicating that the Post-Alen-LNPs prepared via the post-conjugation strategy exhibit superior *in vitro* bone-targeting capability. Direct quantification of Alen ligands on the LNP surface further confirmed that the post-conjugation method loads more Alen ligands onto the LNP surface. This finding is consistent with the trend of binding rates observed in the HAP binding assay, suggesting that the superior bone-targeting performance of the post-conjugation method is primarily attributable to the increased surface ligand density, while improved ligand orientation may also contribute synergistically. This outcome can be attributed to the mechanistic differences between the two modification strategies. In the pre-conjugation method, although a portion of DSPE-PEG-Alen molecules successfully insert into the nanoparticle surface and retain the bone-targeting activity of Alen—thereby conferring a certain degree of targeting functionality—the surface presentation of Alen ligands is susceptible to steric hindrance or phase separation. This results in suboptimal ligand exposure and substantial waste due to ligands being oriented toward the interior of the nanoparticles [42], thereby limiting their targeting capacity. In contrast, the post-conjugation method involves pre-incorporating NHS-activated reactive groups onto the nanoparticle surface, followed by covalent coupling with Alen. This strategy effectively circumvents the issues of ligand burial or misorientation that occur during nanoparticle assembly as a result of steric hindrance or phase separation [43], allowing a greater number of Alen ligands to be displayed on the particle surface in a more controlled manner, thereby achieving higher surface modification efficiency and enabling more effective exertion of their bone-targeting function. The enhanced binding affinity observed in the HAP assay was further corroborated using a more complex ex vivo bone fragment model. When co-incubated with mouse femoral bone fragments, Post-Alen-LNPs exhibited concentration-dependent and specific attachment to the bone surface, whereas unmodified LNPs showed only minimal background binding. This result visually confirms that Alen ligands successfully modified via the post-conjugation method can effectively mediate nanoparticle binding to the native bone matrix, which comprises both organic and inorganic components. Collectively, these *in vitro* findings establish that the post-conjugation strategy enables efficient functionalization of LNPs with Alen, endowing them with robust bone-binding capability [44].

*In vivo* imaging results consistently demonstrated that the Alen modification strategy effectively mediated the selective distribution of LNPs to skeletal tissues, a finding that aligns with the bone affinity observed in the *in vitro* HAP binding assay. Ex vivo organ imaging further revealed that Alen modification did not alter the distribution pattern of LNPs in major organs, indicating that this targeting strategy exhibited favorable tissue selectivity without introducing additional risks of non-specific accumulation. Quantitative analysis showed that Alen-LNPs achieved a 26% increase in bone tissue accumulation compared to unmodified LNPs, a difference that was statistically significant. Although the magnitude of this enhancement was relatively modest, it validated at the *in vivo* level that Alen modification successfully mediates the bone-targeting capability of LNPs. This finding was mutually corroborated by results from the *in vitro* HAP binding assay and the ex vivo bone fragment binding study. Building on this platform, further enhancement of bone-targeting efficiency may be pursued through strategies such as optimizing ligand density, adjusting PEG chain length, or employing multivalent modification approaches [45].

In addition, *in vivo* multi-fluorescence imaging of bone tissue sections revealed clear co-localization of DiD-labeled nanoparticles and GFP protein within bone cells. These findings suggest that after adsorbing to bone tissue through the affinity of Alen for hydroxyapatite in the bone matrix, Alen-LNPs can be effectively internalized by bone-resident cells and successfully deliver mRNA, leading to the translation of functional protein. This result validates the complete capability of the Alen-LNP system—encompassing tissue targeting, cellular uptake, and functional expression—and provides important evidence supporting the feasibility of this system as a gene therapy platform for bone-related diseases. Finally, preliminary biosafety assessment results showed that Alen-LNPs did not induce significant acute systemic toxicity or pathological changes in major organs at the experimental dose, and exhibited low cytotoxicity, indicating good biocompatibility and laying a foundation for its subsequent translational research [46].

This study has certain limitations. For example, Alen exhibits non-selective affinity for hydroxyapatite-rich regions in bone tissue. Future studies may consider integrating “smart” release mechanisms responsive to the pathological bone microenvironment (e.g., acidic pH, specific enzymes) to further enhance treatment precision [47,48]. Additionally, the pharmacokinetic profile and chronic toxicity associated with long-term administration still require in-depth evaluation in large animal models. On this basis, the development of a sequential targeting strategy represents a highly promising research direction. Based on the Alen-LNP platform established in the current work, cell-specific ligands (e.g., peptides targeting osteoblasts or osteoclasts) can be further conjugated to achieve two-stage delivery: first, bone tissue enrichment through the specific binding of Alen to hydroxyapatite; subsequently, ligand-mediated recognition improves the efficiency of mRNA delivery to specific cell subsets. This sequential targeting strategy holds significant potential to broaden the clinical applicability of the current platform.

## 5. Conclusions

In summary, this study successfully established an efficient and stable preparation method for Alen-modified bone-targeting LNPs based on a “post-conjugation” strategy through microfluidic parameter selection. This system exhibited advantages including uniform particle size, stable drug loading, favorable bone-targeting efficiency, and good biocompatibility. It successfully achieved complete verification from *in vivo* tissue targeting to intracellular functional gene delivery. As a proof-of-concept study, this work provides a process reference for the preparation of bone-targeting nanomedicines and establishes a delivery platform with promising translational potential for targeted therapy research of skeletal diseases such as osteoporosis and bone metastases. A critical next step is to evaluate the therapeutic efficacy of this platform in relevant disease models, validating its performance upon loading with therapeutic nucleic acid drugs such as bone-forming or anti-tumor agents, thereby advancing bone-targeted gene therapy toward clinical translation.

## Data Availability

The original contributions presented in this study are included in the article. Further inquiries can be directed to the corresponding author.

## Abbreviations

The following abbreviations are used in this manuscript:

Alen	Alendronate sodium
LNP	Lipid Nanoparticle
mRNA	Messenger RNA
DSPE-PEG	1,2-Distearoyl-sn-glycero-3-phosphoethanolamine-Polyethylene Glycol
NHS	N-Hydroxysuccinimide
HAP	Hydroxyapatite
BMSC	Bone Marrow Mesenchymal Stem Cell
GFP	Green Fluorescent Protein
DiD	1,1′-Dioctadecyl-3,3,3′,3′-Tetramethylindodicarbocyanine Perchlorate
DLS	Dynamic Light Scattering
TEM	Transmission Electron Microscopy
NMR	Nuclear Magnetic Resonance
FT-IR	Fourier Transform Infrared Spectroscopy
H&E	Hematoxylin and Eosin
PDI	Polydispersity Index
EE	Encapsulation Efficiency
CCK-8	Cell Counting Kit-8
ROI	Region of Interest
PBS	Phosphate-Buffered Saline
EDTA	Ethylenediaminetetraacetic Acid
DAPI	4′,6-Diamidino-2-Phenylindole
Pre-Alen-LNP	Pre-conjugated Alendronate-Modified Lipid Nanoparticle
Post-Alen-LNP	Post-conjugated Alendronate-Modified Lipid Nanoparticle
DLin-MC3-DMA	(6Z,9Z,28Z,31Z)-heptatriaconta-6,9,28,31-tetraen-19-yl 4-(dimethylamino)butanoate (ionizable lipid)
DMG-PEG2000	1,2-dimyristoyl-rac-glycero-3-methoxypolyethylene glycol-2000
DSPE-PEG2000-NHS	1,2-distearoyl-sn-glycero-3-phosphoethanolamine-polyethylene glycol 2000-N-hydroxysuccinimide ester
IVIS	In Vivo Imaging System
MWCO	Molecular Weight Cut-off
DSPE-PEG2000-Alen	1,2-distearoyl-sn-glycero-3-phosphoethanolamine-polyethylene glycol 2000-alendronate conjugate
